# Low versus high peripheral oxygen saturation directed oxygen therapy in critically ill patients: a multicenter randomized controlled trial

**DOI:** 10.1002/mco2.70098

**Published:** 2025-02-17

**Authors:** Xiaobo Yang, Xuehui Gao, Xiang Zheng, Xu Zhao, Yanli Liu, Lu Zhang, Junli Sun, Peng Wang, Zhengqin Xu, Ronghua Hu, Hongbin Li, Hong Qi, Yin Yuan, Wei Chen, Jie Liu, Guangqing Huang, Li Yu, Fengsheng Cao, Keke Xin, Min Yu, Xiaoyun Liu, Li Zhang, Siyuan Chang, Xiaojing Zou, Hong Liu, Zhaohui Fu, Huaqing Shu, Yuan Yu, Jiqian Xu, Shiying Yuan, You Shang

**Affiliations:** ^1^ Department of Critical Care Medicine Union Hospital Tongji Medical College Huazhong University of Science and Technology Wuhan China; ^2^ Department of Critical Care Medicine Taihe Hospital Shiyan China; ^3^ Department of Critical Care Medicine Renmin Hospital of Shiyan City Shiyan China; ^4^ Department of Critical Care Medicine The Central Hospital of Wuhan Wuhan China; ^5^ Department of Critical Care Medicine Xiangyang Central Hospital Affiliated Hospital of Hubei University of Arts and Science Xiangyang China; ^6^ General Intensive Care Unit Luoyang Central Hospital Affiliated to Zhengzhou University Luoyang China; ^7^ Department of Critical Care Medicine Renmin Hospital of Yichang City Yichang China; ^8^ Department of Critical Care Medicine Xiangyang No. 1 People's Hospital Affiliated Hospital of Hubei University of Medicine Xiangyang China; ^9^ Department of Critical Care Medicine Hubei Cancer Hospital Wuhan China; ^10^ Department of Critical Care Medicine The First Affiliated Hospital of Zhengzhou University Zhengzhou China

**Keywords:** mechanical ventilation, mortality, oxygen therapy, partial pressure of arterial oxygen, peripheral oxygen saturation, renal replacement therapy

## Abstract

Whether low peripheral oxygen saturation (SpO_2_) directed oxygen therapy is associated with lower mortality in critically ill patients needs further exploration. Adult critically ill patients from 11 intensive care units in China were screened. Participants were randomly assigned to the low SpO_2_ (90%–95%) group or the high SpO_2_ (≥96%) ‐group. The primary outcome was 28‐day all‐cause mortality. The secondary outcomes were hours free from ventilators and from renal replacement therapy (RRT) within 14 days. Note that 857 patients in the low SpO_2_ group and 849 in the high SpO_2_ group were included. In the low SpO_2_ group versus the high SpO_2_ group, the time‐weighted average of the fraction of inspired oxygen (FiO_2_) was significantly lower (33.5 ± 9.7% vs. 39.6 ± 9.3%, *p* < 0.001), and so was the time‐weighted average of SpO_2_ (95.9 ± 1.8% vs. 98.0 ± 1.9%, *p* < 0.001). Within 28 days after randomization, 172 (20.1%) in the low SpO_2_ group and 193 (22.7%) in the high SpO_2_ group died (*p* = 0.180). Ventilator‐free time and RRT‐free time were not significantly different within 14 days. In critically ill patients, low SpO_2_directed oxygen therapy did not decrease 28‐day mortality, 14‐day ventilator‐free time, or 14‐day RRT‐free time.

## Graphical abstract

In critically ill patients, SpO_2_‐directed oxygen therapy did not decrease 28‐day mortality, 14‐day ventilator‐free time, or 14‐day renal replacement therapy (RRT)‐free time in intensive care unit (ICU).

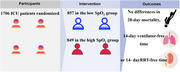

## 1 INTRODUCTION

Oxygen therapy has been employed in clinical settings for over a century.^1^ It has become a standard intervention to administer supplemental oxygen to a wide array of patients experiencing acute and critical illnesses, irrespective of their baseline oxygen saturation levels. The administration of oxygen is often considered a routine part of patient care, with the primary aim of ensuring adequate oxygen delivery to tissues and vital organs. However, despite its widespread use, oxygen therapy may not always confer benefits and can, in fact, be harmful in certain patient populations. Without vigilant monitoring and appropriate titration, the delivery of oxygen can lead to either hypoxia or hyperoxia, both of which pose significant risks to patient health and recovery.^2^

Experimental studies show that the sword of oxygen is double‐edged and either edge cuts. Hypoxia can lead to cell injury and organ failure, as the lack of sufficient oxygen impairs cellular respiration and energy production, ultimately resulting in cellular dysfunction and, if not promptly corrected, organ system collapse.^3^ On the other hand, hyperoxia may also cause cell, tissue, or organ injury due to enhanced oxidative stress and inflammation.^4^, ^5^ The excess of oxygen can lead to the formation of reactive oxygen species, which are highly reactive molecules capable of damaging cellular components, including lipids, proteins, and DNA.^6^ This oxidative damage can trigger apoptosis, or programmed cell death, and can also activate inflammatory pathways, leading to a pro‐inflammatory state that can exacerbate tissue injury and impede the healing process. The promotion of oxygen free radical generation overwhelms the body's antioxidant defenses, leading to the activation of both anti‐inflammatory and pro‐inflammatory factors.^7^, ^8^ This dual activation can create a detrimental environment for cells, tipping the balance toward cell death through apoptosis or necrosis; moreover, the change in the internal immune state induced by hyperoxia can lead to an increased risk of infection.^6^, ^7^, ^8^ The implications of these findings are significant for the clinical management of patients requiring oxygen therapy.

To optimize oxygen therapy, clinical studies have been conducted. Some studies have demonstrated that excessive oxygen therapy may worsen outcomes in patients with conditions such as myocardial infarction^9^ and resuscitation from cardiac arrest.^10^ For general patients in the intensive care unit (ICU), retrospective studies showed that hypoxemia was associated with an increased risk of ventilator ‐associated pneumonia^11^ and mortality.^12^, ^13^, ^14^ Through retrospective analyses of data from two electronic medical record databases, van den Boom et al. found that the optimal saturation of pulse oximetry (SpO_2_) for critically ill patients was between 94% and 98%.^15^ In the 2016 Effect of Conservative versus Conventional Oxygen Therapy on Mortality Among Patients in an Intensive Care Unit (Oxygen‐ICU) study, Girardis et al. showed that ICU mortality was lower in the conservative oxygen therapy group (target arterial oxyhemoglobin saturation between 94% and 98% or partial pressure of arterial oxygen (PaO_2_) between 70 and 100 mmHg) than in the conventional oxygen group (target arterial oxyhemoglobin saturation ≥ 97%).^16^ However, the sample size of this study was relatively small. In 2017, the British Thoracic Society Emergency Oxygen Guideline recommended that the SpO_2_ of most acute patients should be set to 94%–98%.^17^ However, it has been reported that the target value of the British Thoracic Society guidelines will still lead to a large number of hyperxemia.^18^ The 2018 clinical practice guidelines for acute patients suggest that the SpO_2_ of patients receiving oxygen therapy should not exceed 96%, which applies to almost all hospitalized patients.^19^ Large‐scale randomized studies are needed to guide oxygen therapy in general ICU patients.

In recent years, several such studies have been reported: the Intensive Care Unit Randomized Trial Comparing Two Approaches to Oxygen Therapy (ICU‐ROX) study,^20^ the Pragmatic Investigation of Optimal Oxygen Targets Trial (PILOT) study,^21^ the Liberal Oxygenation versus Conservative Oxygenation in ARDS (LOCO_2_) study,^22^ the Handling Oxygenation Targets in the ICU (HOT‐ICU) study,^23^ the Optimal Oxygenation in the Intensive Care Unit (O_2_‐ICU) study,^24^ and the Conservative versus Liberal Oxygenation Targets in Intensive Care Unit Patients (ICONIC) study.^25^ Almost concurrently with these studies, we were conducting the Peripheral Oxygen Saturation Directed Oxygen Therapy (POSDOT) study to determine whether lower SpO_2_ targets could provide better outcomes for critically ill patients than higher SpO_2_ targets.

## 2 RESULTS

### 2.1 Characteristics of the patients

**FIGURE 1:**
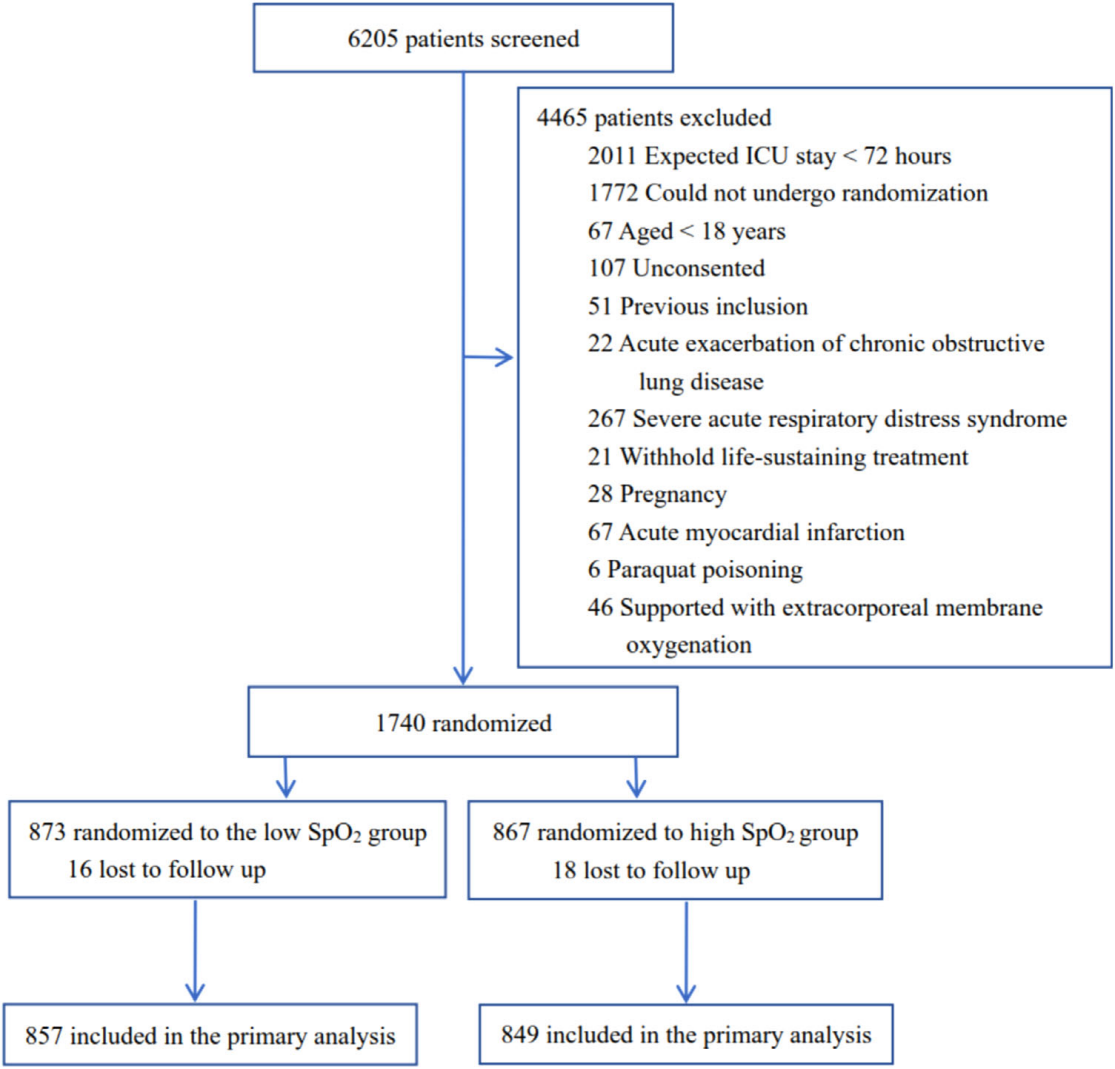
Flowchart of patient inclusion.

From May 2019 to May 2022, we enrolled 1740 patients in 11 general ICUs from 10 tertiary hospitals in China. However, the enrollment was heavily influenced by the COVID‐19 pandemic, we decided to stop the study, and our decision was approved by the ethics committee of our institution. Loss to follow‐up occurred in 34 patients, which left an intention‐to‐treat (ITT) population of 1706, with 857 assigned to the low SpO_2_ group and 849 to the high SpO_2_ group (Figure 1). Their mean age was 59.7 ± 15.6 years, 1087 (63.7%) were male, and 1138 (66.7%) were medical patients (Table 1). At admission, 479 (28.1%) patients and 1067 (62.5%) were experiencing shock and respiratory failure, respectively. Their Acute Physiology and Chronic Health Evaluation II (APACHE II) score was 15.9 ± 7.3, and Charlson Comorbidity Index was 1 [0, 3]. The interval between ICU admission and randomization was 17.2 ± 13.6 h. A total of 1445 (84.7%) patients had an ICU stay of no less than 72 hours.

**TABLE 1:** Baseline characteristics of the patients included.

Demographic	All patients (*n* = 1706)	Low SpO_2_ group (*n* = 857)	High SpO_2_ group (*n* = 849)
Age (years)	59.7 ± 15.6	60.2 ± 15.1	59.2 ± 16.0
Sex: male (%)	1087 (63.7%)	548 (63.9%)	539 (63.9%)
Admission			
Medical	1138 (66.7%)	566 (66.0%)	572 (67.4%)
Planned surgery	165 (9.7%)	82 (9.6%)	83 (9.8%)
Emergency surgery	403 (23.6%)	209 (24.4%)	194 (22.9%)
Organ failure			
Shock	479 (28.1%)	237 (27.7%)	242 (28.5%)
Septic	299 (17.5%)	145 (16.9%)	154 (18.1%)
Hypovolemia	137 (8.0%)	72 (8.4%)	65 (7.7%)
Cardiac	35 (2.1%)	16 (1.9%)	19 (2.2%)
Obstructive	8 (0.5%)	4 (0.5%)	4 (0.5%)
APACHE II score	15.9 ± 7.3	15.9 ± 7.4	16.0 ± 7.2
Charlson Comorbidity Index	1 [0, 3]	1 [0, 2]	1 [0, 3]

*Note*: Data were presented as mean ± standard deviation, count (percentage) or median [interquartile range].

Abbreviations: APACHE II, Acute Physiology and Chronic Health Evaluation II; ICU, intensive care unit; SpO_2_, peripheral oxygen saturation.

### 2.2 Mechanical ventilation and oxygen support

There were no significant differences between the low SpO_2_ group and the high SpO_2_ group in the percentages of patients receiving mechanical ventilation at randomization (respectively, 62.5% vs. 63.3%, *p* = 0.762) and ever during the study period (respectively, 68.1% vs. 69.4%, *p* = 0.583). Compared to the low SpO_2_ group, a higher time‐weighted average FiO_2_ (39.6 ± 9.3% vs. 33.5 ± 9.7%, *p* < 0.001) led to a higher time‐weighted average SpO_2_ (98.0 ± 3.6% vs. 95.9 ± 1.8%, *p* < 0.001) in the high SpO_2_ group (Table 2). The distribution of time‐weighted average FiO_2_ and time‐weighted average SpO_2_ are presented in Table 3.

**TABLE 2:** Mechanical ventilation and oxygen support.

Treatment	Low SpO_2_ group (*n* = 857)	High SpO_2_ group (*n* = 849)	*p*‐value
Mechanical ventilation at randomization	536 (62.5%)	537 (63.3%)	0.762
Ever receiving mechanical ventilation during the study	584 (68.1%)	589 (69.4%)	0.583
**Oxygen support**			
Time‐weighted FiO_2_ (%)	33.5 ± 9.7	39.6 ± 9.3	<0.001
Time‐weighted SpO_2_ (%)	95.9 ± 1.8	98.0 ± 1.9	<0.001
ABG data			
**Before inclusion**			
FiO_2_ from ABG^a^ (%)	43.0 ± 14.1	43.4 ± 13.8	0.721
PaO_2_ from ABG^a^ (mmHg)	102.4 ± 36.6	105.9 ± 38.1	0.135
**After inclusion**			
Time‐weighted FiO_2_ from ABG^b^ (%)	33.9 ± 12.8	41.3 ± 12.4	<0.001
Time‐weighted PaO_2_ from ABG^b^ (mmHg)	87.6 ± 14.4	100.6 ± 19.3	<0.001

*Note*: Data were presented as mean ± standard deviation or count (percentage).

Abbreviations: ABG, arterial blood gas analysis; FiO_2_, fraction of oxygen; PaO_2_, partial pressure of oxygen; PEEP, positive end‐expiratory pressure; SpO_2_, peripheral oxygen saturation.

^a^ Data from 506 patients in the low SpO_2_ group and 500 patients in the high SpO_2_ group.

^b^ Data from 598 patients in the low SpO_2_ group and 597 patients in the high SpO_2_ group.

**TABLE 3:** Outcomes.

Outcomes	Low SpO_2_ group (*n* = 857)	High SpO_2_ group (*n* = 849)	*p*‐value
Primary outcome			
28‐Day mortality	172 (20.1%)	193 (22.7%)	0.180
Secondary outcomes			
Ventilator free time in 14 days (h)	59 [24, 105]	63 [24, 114]	0.737
Renal replacement free time in 14 days (h)	120 [74, 195.5]	120 [75, 200]	0.670

*Note*: Data were presented as count (percentage) or median [interquartile range].

Abbreviation: SpO_2_, peripheral oxygen saturation.

In patients with data of FiO_2_ and PaO_2_ from arterial blood gas analysis, before inclusion, FiO_2_ (43.0 ± 14.1% in the low SpO_2_ group vs. 43.4 ± 13.8% in the high SpO_2_ group, *p* = 0.721) and PaO_2_ (102.4 ± 36.6 mmHg in the low SpO_2_ group vs. 105.9 ± 38.1 mmHg in the high SpO_2_ group, *p* = 0.135) were not different between the two groups (Table 2). After inclusion, time‐weighted average FiO_2_ and time‐weighted average PaO_2_ were significantly lower in tlow SpO_2_ group than in the high SpO_2_ group (33.9 ± 12.8% vs. 43.4 ± 13.8%, *p* < 0.001 and 87.6 ± 14.4 mmHg vs. 100.6 ± 19.3 mmHg, *p* < 0.001, respectively).

### 2.3 Outcomes

**FIGURE 2:**
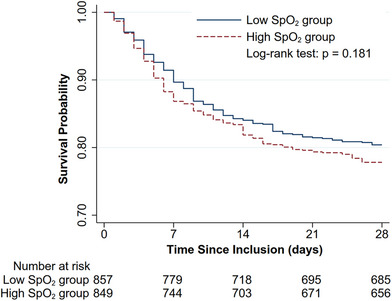
Kaplan–Meier curves demonstrating the survival probability of the low SpO_2_ group and the high SpO_2_ group. SpO_2_, peripheral oxygen saturation.

Within 28 days after randomization, 172 (20.1%) patients in the low SpO_2_ group and 193 (22.7%) in the high SpO_2_ group died (risk ratio [RR] 0.88, 95% confidence interval [CI] [0.74–1.06], *p* = 0.180) (Table 3). The log‐rank test of the probability of survival between the two groups since randomization was not different either (*p* = 0.181, Figure 2). As for the secondary outcomes, ventilator‐free time (59 [24, 105] h in the low SpO_2_ group vs. 63 [24, 114] h in the high SpO_2_ group, *p* = 0.737) and renal replacement therapy (RRT)‐free time (120 [74, 195.5] h in the low SpO_2_ group vs. 120 [75, 200] h in the high SpO_2_ group, *p* = 0.670) in 14 days were not different (Table 3). Subgroup analyses based on baseline characteristics showed a borderline non‐significant decrease in 28‐day mortality in female patients, and a borderline significant decrease in 28‐day mortality in patients with a Charlson Comorbidity Index greater than 0. Univariate and multivariate Cox regression analyses showed age, shock at mission, and respiratory failure at admission were risk factors for death (Table S1). In either analysis, high SpO_2_ was not a risk factor for death.

## 3 DISCUSSION

Our multicenter, randomized clinical trial showed that low SpO_2_‐directed oxygen therapy did not decrease 28‐day mortality, 14‐day ventilator‐free time, or 14‐day RRT‐free time in critically ill patients.

SpO_2_ is employed routinely in clinical practice and commonly in trials exploring the correlation between oxygen therapy and clinical outcomes in critically ill patients. Based on data from more than 30,000 patients from two electronic medical record databases, van den Boom and colleagues revealed that compared patients with 40% of the time SpO_2_ between 94% and 98%, patients with 80% of the time SpO_2_ within the same range had a nearly halved risk of hospital death in a retrospective study.^15^ Although, in our study, the time‐weighted average SpO_2_ was between 94% and 98% in the low SpO_2_ group, we did not find a difference in mortality in 28 days, ventilator‐free time in 14 days, or RRT‐free time in 14 days compared with the high SpO_2_ group. Our findings are consistent with findings from the ICU‐ROX study^20^ and the PILOT study.^21^ The ICU‐ROX investigators randomized 1000 adult mechanically ventilated patients into the conservative‐oxygen group (97% as upper limit of SpO_2_ and FiO_2_ as low as possible to 21%) and the usual oxygen group (no upper limit of SpO_2_ and FiO_2_ above 30%), and they found no difference in ventilator‐free days or 180‐day mortality.^20^ In the PILOT study, Semler and colleagues assigned mechanically ventilated patients into three groups based on SpO_2_ target, the low SpO_2_ group (88%–92%), the intermediate SpO_2_ group (92%–96%), and the high SpO_2_ group (96%–100%); they found no difference in ventilator‐free days or 28‐day mortality among these groups.^21^

Other two commonly used targets in oxygenation studies in ICUs are PaO_2_ and arterial oxyhemoglobin saturation (SaO_2_). Compared with patients with SaO_2_ ≥ 97%, Girardis and colleagues found that patients with SaO_2_ between 94% and 98% or PaO_2_ of 70–100 mmHg had a significantly reduced chance of ICU death from 20.2% to 11.6%, of shock from 10.6% to 3.7%, and of bacteremia from 10.1% to 5.1%.^16^ However, Oxygen‐ICU was an early‐terminated single‐center randomized controlled study with only 480 patients included. These findings were not replicated by the LOCO_2_ study^22^ and the HOT‐ICU study.^23^ In the LOCO_2_ study, Barrot and colleagues included 205 patients with acute respiratory distress syndrome and found no difference in 28‐day mortality between patients with PaO_2_ from 55 to 70 mmHg and patients with PaO_2_ from 90 to 105 mmHg.^22^ In the HOT‐ICU study on adult patients with acute respiratory failure, 1441 were randomized into the lower oxygenation group (target PaO_2_ 60 mmHg) and 1447 into the higher oxygenation group (target PaO_2_ 90 mmHg), and no difference in 90‐day mortality was found.^23^

The difference between mean/median FiO_2_ in the high target group and mean/median FiO_2_ in the low target group varies. It increases from 3% in the Oxygen‐ICU study,^16^ approximately 5% in the ICU‐ROX study,^20^ approximately 6% in our study, approximately 10% in the O_2_‐ICU study^24^ and the LOCO_2_ study,^22^ approximately 12.6% in the ICONIC study,^25^ and approximate 15% in the PILOT study^21^ and the HOT‐ICU study.^23^ The different differences in FiO_2_ between the high target group and the low target group in these studies make it difficult to conduct a meta‐analysis.

In the low SpO_2_ group/conservative group, the actual SpO_2_ levels were commonly higher than the predefined upper limit of the SpO_2_ target. In the low SpO_2_ group/conservative group, the time‐weighted average of SpO_2_ was 93.4% in the study of Panwar and colleagues,^26^ 95.9% in our study, and approximately 94% in the PILOT study^21^; the predefined upper limit of the SpO_2_ target was 92%, 95%, and 92%, respectively.

Even in pO_2_‐targeted study, the actual SpO_2_ levels were commonly higher than the anticipated SpO_2_ levels in the lower target group. The actual SpO_2_ was approximately 93% in the LOCO_2_ study^22^ and in the HOT‐ICU study,^23^ and the anticipated SpO_2_ levels were 88%–92% and approximately 90%, respectively. Whether compliance with the study protocol will make a difference is unknown. The bottom line is an extremely large number of participants should be included to make a firm conclusion on whether there will be a significant difference in mortality between conservative oxygen therapy and liberal oxygen therapy in critically ill patients, and we are hoping that the Mega‐ROX trial (ACTRN12620000391976) will shed some light on the question.

The strengths of our study lie in the pragmatic protocol allowing us to maintain routine practice except for oxygenation targets and differences in SpO_2_ and FiO_2_ between the two groups. However, our study had some limitations. First, our study stopped prematurely because of the COVID‐19 pandemic. However, it was unlikely the planned sample size would make a difference in the primary outcome. Second, a large proportion of patients screened were not randomized. For one thing, one liaison at each study center was asked to screen as many patients as possible, and patients admitted into the ICU during weekends or holidays were easily categorized as being unable to be randomized. For another, the plan to include patients with an ICU stay of at least 72 hours aimed at a longer duration of oxygen treatment in the ICU, but it also led to the unnecessary exclusion of potentially eligible patients with an uncertain length of ICU stay at the time of screening. The screening process is difficult to regulate and monitor.^27^ Third, the SpO_2_ target was not achieved in the low SpO_2_ group, which may attenuate the treatment effect. Fourth, data on arterial blood analysis were not mandatorily collected. The relationship between arterial SpO_2_ and PaO_2_ is not linear, and compared with the PaO_2_ target, SpO_2_ targe is a relatively less precise titration of oxygenation. But SpO_2_ has the advantage of real‐time continuous monitoring. Since no compensation was given to the patients, our ethics committee deemed it would be immoral to conduct arterial blood gas analyses only for the study. In the present trial, PaO_2_ and FiO_2_ were obtained from arterial blood gas analyses at the discretion of study physicians. Because pO_2_ was measured at different times and could not be measured continuously, SpO_2_ was used to titrate oxygen.^23^, ^25^ In the ICONIC study, only 68.3% and 52.9% of patients in the low oxygenation group and the high oxygenation group, respectively, had 50% or more of pO_2_ measurements within the predetermined ranges.^25^ This information was not available in the HOT‐ICU study.^26^ The SpO_2_ values concomitant to pO_2_ measurements of only three to four times per day were sufficient to differentiate between the low oxygenation group and the high oxygenation group.^25^ Fifth, all participating study centers were tertiary hospitals from China, which would restrict the generalizability of this study. Sixth, data on ischemic events, including myocardial infarction, ischemic stroke, and intestinal ischemia, were unavailable. Seventh, although the free time in hours of respiratory and of renal support with machines wwas planned and explored as possible outcome measures for future trials, we failed to find differences in these two aspects. For convenience, a fixed time frame of 14 days for the secondary outcomes was used, which was the same as the duration of the oxygen fraction manipulation in the present study. Secondary outcomes within 14 days of enrollment, such as ventilator‐free days, were also used by Gelissen and colleagues in the O_2_‐ICU study.^24^

## 4 CONCLUSION

In critically ill patients, the low SpO_2_‐directed oxygen therapy did not lead to a lower 28‐day mortality, a longer duration free from ventilator, or a longer duration free from RRT in 14 days.

## 5 MATERIALS AND METHODS

### 5.1 Study design

POSDOT study was a multicenter randomized controlled trial, conducted in accordance with the Declaration of Helsinki and the International Conference on Harmonisation Good Clinical Practice. The protocol (Supporting Information 1) and oral or written informed consent were approved by the ethics committee of each institution, and informed consent was obtained from the patients or legal representatives.

### 5.2 Patients

All patients aged greater than or equal to 18 years were eligible if they were admitted to the ICU with an expected length of stay no less than 72 hours. Exclusion criteria were as follows: pregnancy, acute exacerbation of chronic obstructive pulmonary disease, severe acute respiratory distress syndrome (defined as PaO_2_/the fraction of oxygen (FiO_2_) ≤ 100 mmHg and PEEP ≥ 10 cmH_2_O while on a ventilator), acute myocardial infarction, paraquat poisoning, receiving extracorporeal membrane oxygenation, withholding or withdrawal of life‐sustaining treatment, failure to screen within 48 h since admission to ICU, refusal to be included, prior participation in this study or other interventional studies within 3 months, and any other reasons deemed inappropriate by the study doctors.

### 5.3 Randomization and intervention

Enrolled patients were randomized in a 1:1 ratio to the low SpO_2_ group (SpO_2_ target set at 90%–95%) or the high SpO_2_ group (SpO_2_ target set at 96%–100%) according to a computer‐generated sequence concealed in closed opaque envelopes based on random block sizes of 2, 4, 6, 8, or 10 with stratification on each participating site.

This was an open‐label trial to account for the impossibility of concealing the treatment assignments. For patients mechanically ventilated, if SpO_2_ was not within the predefined range, FiO_2_ was adjusted by 0.05 (absolute value) every 30 min. In the low SpO_2_ group, when SpO_2_ was between 90% and 95%, FiO_2_ was administered as low as possible to 21%. In the high SpO_2_ group, FiO_2_ was adjusted to reach SpO_2_ higher than 96% as long as FiO_2_ ≥ 30%. For patients not mechanically ventilated, the flow of oxygen was titrated every 30–60 min. For patients with severely poor peripheral perfusion when SpO_2_ was unattainable, saturation of oximetry from arterial blood analysis was used. The SpO_2_ alarms on all monitors were set accordingly. Clear signs were written or hung at the bedside of enrolled patients. Nurses and physicians caring for these patients adjusted FiO_2_ to achieve the target SpO_2_. FiO_2_ and SpO_2_ were recorded every 6 h. The intervention was stopped at 14 days, death, or ICU discharge, whichever came first.

In case of a procedure, such as endotracheal intubation, fibroscopy or tracheotomy, FiO_2_ adjustment was determined by the treating clinicians. We recommended following the protocol as much as possible and returning to the protocol as soon as possible. If a patient's condition deteriorates, the treating clinicians could change the SpO_2_ target if they feel it was in the best interest of the patient. All other treatments, including tracheal intubation, ventilator setting except FiO_2_, vasoactive drugs, imaging examination, and microbiological specimen collection, referred to the routine of each institution.

### 5.4 Data collection

A standardized case report form was used to collect data. At enrollment, we collected demographic data, type of admission (medical, elective surgical or emergency surgical), APACHE II score, shock,^26^ respiratory failure (PaO_2_/ FiO_2_ < 300 mmHg), and Charlson Comorbidity Index. Time‐weighted averages of FiO_2_ and SpO_2_ were calculated.^16^, ^27^ Arterial blood gas analysis was performed at the discretion of the treating physicians according to the routine of each institution. Time‐weighted averages of FiO_2_ and pO_2_ from arterial blood gas analysis were calculated in the same way.

### 5.5 Outcomes

The primary outcome was 28‐day all‐cause mortality. Follow‐up phone calls were made after discharge to confirm whether patients were alive 28 days after inclusion. The secondary outcomes were hours of free time from ventilators and from RRT within 14 days after inclusion when patients were still in the ICU included in the present study.

### 5.6 Statistical analysis

On the basis of previous data from our pilot study with a 28‐day mortality of 30%, the originally planned sample size was 2148 patients to detect an absolute difference in mortality of 6% between the low SpO_2_ group and the high SpO_2_ group (*ɑ* = 0.05, *β* = 80%).

All analyses were conducted following the ITT principle. Continuous data were presented as the mean ± standard deviation (SD) or median interquartile range. Unpaired Student's *t*‐test was used to evaluate differences between the two groups. Otherwise, the Mann–Whitney *U* test was used. Categorical data were expressed as counts (percentages) and analyzed using the chi‐square test. Mortality over time was assessed with Kaplan–Meier analysis and log‐rank test. The exploratory post hoc subgroup analyses were conducted to assess whether there was heterogeneity in treatment effects on the primary outcome. The RR with 95% CI was used for the presentation of the mortality rate ratio between the two groups when appropriate. Univariate and multivariate Cox regression analyses were used to explore the risk factors for 28‐day mortality. A *p*‐value < 0.05 was considered statistically significant. We used Stata/IC 15.1 software (StataCorp) for statistical analysis.

## AUTHOR CONTRIBUTIONS

*Drafting of the manuscript*: Xiaobo Yang, Xuehui Gao, Xiang Zheng, Xu Zhao, Yanli Liu, Lu Zhang, Junli Sun, Peng Wang, Zhengqin Xu, Ronghua Hu, and Hongbin Li. *Critical revision of the manuscript*: Hong Qi, Yin Yuan, Wei Chen, Jie Liu, Guangqing Huang, Fengsheng Cao, and Keke Xin. *Administrative and technical support*: Li Yu, Min Yu, Xiaoyun Liu, Li Zhang, Siyuan Chang, Xiaojing Zou, Hong Liu, Zhaohui Fu, Huaqing Shu, Yuan Yu, Jiqian Xu, Shiying Yuan, and You Shang. *Acquisition, analysis, and interpretation of data*: All Authors. All authors have read and approved the final manuscript.

## CONFLICT OF INTEREST STATEMENT

The authors declare no conflicts of interest.

## ETHICS STATEMENT

This study was approved by the ethics committee of Tongji Medical College (No. 2017‐S024) and ethics committees of other study sites. Informed consent was obtained from all participants. This trial was registered on Clinicaltrials.gov (NCT02999932).

## Supporting information



Supporting Information

## Data Availability

Data will be made available 2 years after publication upon reasonable request by researchers who provide a methodologically sound proposal and whose use of the data has been approved by the study authors and the ethics committee of the corresponding authors.
