# Supplementary material for: Low versus high peripheral oxygen saturation directed oxygen therapy in critically ill patients: a multicenter randomized controlled trial
Source: MedComm (2020). 2025 Feb 17;6(3):e70098. doi: 10.1002/mco2.70098 (PMC11831183; doi:10.1002/mco2.70098)
Supplement: Supplementary file 1 — Supporting Information [file MCO2-6-e70098-s001.docx]

**Effect of Low vs High Pulse Oxygen Saturation (SpO_2_) Directed Oxygen Therapy on Mortality among Critically Ill Patients (POSDOT)**

**Background**

Oxygen therapy is widely used in clinical practice and is essential in the treatment of patients with absolute or relative hypoxia (1,2). However, hyperoxia may lead to organ damage including lung injury (3,4). Recent in vivo and in vitro animal experiments and human studies have shown that high concentrations of oxygen alter physiological processes in normal cells: promoting oxygen free radical generation, activating apoptosis, anti- and pro-inflammatory factors leading to cell death, and alterations in the intrinsic immune status leading to an increased risk of infection (5-7).

Hyperoxia is likely to affect the prognosis of intensive care unit (ICU) patients. In the AVOID study, Meyhoff et al. found that oxygen therapy resulted in more cardiac injury in patients with acute ST-segment elevation and no hypoxia (8). Kilgannon et al. found that hyperoxia was associated with increased in-hospital morbidity and mortality in patients with successfully resuscitated cardiac arrest (9). Recently, a retrospective study in the Netherlands found that in mechanically ventilated patients, high levels of inhaled oxygen and high partial pressure of oxygen were associated with increased ICU morbidity and mortality (10).

However, there are not enough clinical studies to determine how high a concentration of inhaled oxygen is safe to maintain adequate tissue oxygen supply, much less how high and for how long it causes cellular damage. The Volume and Pressure Aspects of Mechanical Ventilation in Patients with Acute Respiratory Distress Syndrome (ARDS) study (11) recommended the use of the lowest inhaled oxygen concentration to maintain normoxia (12). However, in fact, oxygen therapy in the ICU is very heterogeneous, as confirmed by a research study conducted in a Canadian ICU (13). Some experts recommend lower concentrations of oxygen therapy under conditions of maintaining tissue oxygen supply and call for clinical studies to assess the benefits or disadvantages of permissive hypoxia or conservative oxygen delivery strategies (14). Recently, in a small-sample study, Panwar et al. found that in mechanically ventilated ICU patients, a conservative oxygen strategy (SpO_2_ 88-92%) was feasible and did not increase morbidity or mortality compared with a liberal oxygen strategy (SpO_2_ ≥96%) (15).A larger-sample randomized controlled study by Girardis et al. published in JAMA found that compared with conventional oxygen therapy Compared with conventional oxygen therapy (SpO_2_ ≥97%), controlled oxygen therapy (SpO_2_ target 94%-98%, or arterial partial pressure of oxygen 70-100 mmHg) led to a reduction in ICU morbidity and mortality (16). However, this was a single-center study, and the study had to be terminated early because of earthquakes after enrolling only 480 patients, necessitating an unplanned interim analysis. Girardis and other experts have called for a large-sample, multicenter study to assess the safety of controlled oxygen therapy and its impact on morbidity and mortality (16,17).

**Objectives**

The main objective of the study is to determine the mortality rates of patients within 28 days after being included. Additionally, the study also looks at the amount of time the patients were not on ventilators or undergoing renal replacement therapy within 14 days after being included as secondary objectives.

**Inclusion criteria and exclusion criteria**

Patients who are 18 years old or above and have given their consent are included if they are admitted to the ICU and are expected to stay there for more than 72 hours.

Patients meeting any of the following conditions will not be considered for participation: previous inclusion, those experiencing acute exacerbation of chronic obstructive disease, severe acute respiratory distress syndrome (as defined by a PaO_2_/FiO_2_ ratio less than or equal to 100mmHg and PEEP levels of at least 10cmH_2_O), those who have chosen to forego life-sustaining treatment, physician failure to screen within the initial 72 hours following admission, expectant mothers, acute myocardial infarction, paraquat poisoning, and those currently receiving or planning to receive extracorporeal membrane oxygenation (ECMO).

**Randomization and group management**

Patients enrolled will be randomly assigned to either the low SpO_2_ group (SpO_2_ goal of 90-95%) or the high SpO_2_ group (SpO_2_ goal of 96-100%). The assignments are made according to a computer-generated sequence hidden in sealed opaque envelopes, using random block sizes of 2, 4, 6, 8, or 10, and stratified upon each participating institution.

The low SpO_2_ group has the FiO_2_ set as low as possible, while the high SpO_2_ group has it no lower than 30%. If SpO_2_ couldn't be measured, arterial blood gas is used. The SpO_2_ alarm on all monitors will be set accordingly. Clear signs are written or hung at the bedside of enrolled patients. Nurses and physicians caring for these patients adjust FiO_2_ to achieve the target SpO_2_.In critical situations, like intubation or hemodynamic collapse, FiO_2_ could be temporarily raised to 100%. If severe adverse events occur in the low SpO_2_ group, the patient could be withdrawn. Other actions are performed according to institutional protocols, and the treating team adjust the FiO_2_ accordingly, while the included patients are unaware of their treatment group.

**Data collection**

Data are gathered using a standardized case report form. At enrollment, we gather information on the patient's demographics, type of admission (medical, elective, or emergency surgical), APACHE II score, shock, respiratory failure (PaO_2_/FiO_2_ 300mmHg), and Charlson comorbidity score. FiO_2_ and SpO_2_ are recorded every six hours up to 14 days, death, or ICU discharge, whichever comes first. To confirm the patients' survival, follow-up phone calls will be conducted post-discharge.

**Statistical analysis**

Based on data obtained from our pilot study, wherein a 30% mortality rate was recorded over a period of 28 days, we plan to have a sample size of 2148 patients to determine a difference of 6% in 28-day mortality between the low SpO_2_ group and the high SpO_2_ group with ɑ = 0.05 and β= 80%. FiO_2_ and SpO_2_ time-weighted averages are employed for statistical analysis. In the same way, the time-weighted averages of FiO_2_ and pO_2_ from the arterial blood gas analysis are computed in the same way. Following the intention-to-treat (ITT) principle, all analyses were performed. Continuous data were displayed as the median interquartile range or mean standard deviation. To assess differences between the two groups, an unpaired Student's t-test was employed. The Mann-Whitney U test was applied if not. The chi-square test was used to examine categorical data that were presented as counts (percentages). With the help of the log-rank test and Kaplan-Meier analysis, mortality over time was evaluated. Statistical significance was defined as a p value less than 0.05.

**Data attribution and publication of findings**

Considering that this study is designed to add to the knowledge on oxygen therapy and to assess the impact of oxygen therapy on critically ill patients in ICUs, and that patients and their families volunteer to participate in this study, the investigators feel that they should ensure wider dissemination of the data and results in a faithful and responsible manner.

Researchers have the right to publish the research protocol and preliminary results in seminars, academic conferences, in academic journals, in dissertations, and ultimately in formal publications in academic journals.

**Reference**

1. Martin DS, GrocottMPW. Oxygen therapy in critical illness: precise control of arterial oxygenation and permissive hypoxemia. Crit Care Med. 2013; 41(2):423-432.

2. O’Driscoll BR, Howard LS, Davison AG; British Thoracic Society. BTS guideline for emergency oxygen use in adult patients. Thorax. 2008; 63(suppl 6):vi1-vi68.

3. Nash G, Blennerhassett JB, Pontoppidan H. Pulmonary lesions associated with oxygen therapy and artificial ventilation. N Engl J Med 1967; 276:368–374.

4. Crapo JD, Hayatdavoudi G, Knapp MJ, Fracica PJ, Wolfe WG, Piantadosi CA: Progressive alveolar septal injury in primates exposed to 60% oxygen for 14 days. Am J Physiol 1994, 267:L797-L806.

5. Dieperink HI, Blackwell TS, Prince LS. Hyperoxia and apoptosis in developing mouse lung mesenchyme. Pediatr Res 2006; 59:185–190.

6. Bhandari V, Elias JA. Cytokines in tolerance to hyperoxia-induced injury in the eveloping and adult lung. Free Radic Biol Med 2006; 41:4–18.

7. Baleeiro CE, Wilcoxen SE, Morris SB, et al. Sublethal hyperoxia impairs pulmonary innate immunity. J Immunol 2003; 171:955–963.

8. Stub D, Smith K, Bernard S, et al; AVOID Investigators. Air versus oxygen in ST-segment-elevationmyocardial infarction. Circulation. 2015; 131(24): 2143-2150.

9. Kilgannon JH, Jones AE, Shapiro NI, et al; Emergency Medicine Shock Research Network (EMShockNet) Investigators. Association between arterial hyperoxia following resuscitation from cardiac arrest and in-hospital mortality. JAMA. 2010; 303(21): 2165-2171.

10. de Jonge E, Peelen L, Keijzers PJ, Joore H, de Lange D, van der Voort PHJ, Bosman RJ, de Waal RAL, Wesselink R, de Keizer NF. Association between administered oxygen, arterial partial oxygen pressure and mortality in mechanically ventilated intensive care unit patient. Critical Care 2008;12: R156.

11. Brower RG, Lanken PN, MacIntyre N, et al. Higher versus lower positive endexpiratory pressures in patients with the acute respiratory distress syndrome.N Engl J Med 2004;

351:327–336.

12. The Acute Respiratory Distress Syndrome Network: Ventilation with lower tidal volumes as compared with traditional tidal volumes for acute lung injury and the acute respiratory distress syndrome. N Engl J Med 2000, 342: 1301-1308.

13. Mao C, Wong DT, Slutsky AS, Kavanagh BP. A quantitative assessment of how Canadian intensivists believe they utilize oxygen in the intensive care unit. Crit Care Med 1999

; 27(12):2806-11.

14. Altemeier WA, Sinclair SE. Hyperoxia in the intensive care unit: why more is not always better. Current Opinion in Critical Care 2007; 13: 73-78.

15. Panwar R, Hardie M, Bellomo R, et al; CLOSE Study Investigators; ANZICS Clinical Trials Group. Conservative versus liberal oxygenation targets for mechanically ventilated patients: a pilot multicenter randomized controlled trial. Am J Respir Crit Care Med. 2016; 193(1):43-51.

16. Girardis M, Busani S, Damiani E, et al. Effect of conservative vs conventional oxygen therapy on mortality among patients in an intensive care unit: the Oxygen-ICU randomized clinical trial. JAMA. 2016; 316(15):1583-1589.

17. Ferguson N. Oxygen in the ICU: Too Much of a Good Thing? JAMA. 2016; 316(15):1553-1554.

**Trial modification**

**Exclusion criteria modification**

A time frame of 12 hours after admission to the ICU without screening as an exclusion criterion was considered too strict and was extended to 48 hours by agreement of all investigators.

**Data collection modification**

PaO_2_ and FiO_2_ from arterial blood gas analysis at inclusion and after inclusion were recommended to collect during the latter part of the study.

**Statistical plan modification**

Univariate and multivariate Cox regression analyses were used to examine the risk factors associated with mortality at 28 days.

**Supplementary table 1. Univariate and Multivariate Cox Regression Analysis of Risk Factors for Death in 28 Days**

|  | Univariate |  | Multivariate |  |
| --- | --- | --- | --- | --- |
| Variable | Hazard ratio [95% CI] | p value | Hazard ratio [95% CI] | p value |
| Age (years) | 1.02 [1.01, 1.02] | < 0.001 | 1.01 [1.00, 1.02] | < 0.001 |
| Female sex | 0.97 [0.78, 1.20] | 0.753 | 0.95 [0.77, 1.18] | 0.661 |
| Shock at admission | 1.55 [1.24, 1.92] | < 0.001 | 1.46 [1.17, 1.82] | < 0.001 |
| Respiratory failure at inclusion | 1.67 [1.32, 2.10] | < 0.001 | 1.60 [1.26, 2.01] | < 0.001 |
| High SpO_2_ group | 1.15 [0.94, 1.42] | 0.185 | 1.17 [0.95, 1.44] | 0.147 |

CI: confidence interval; SpO_2_: peripheral oxygen saturation.
